# Beyond respiratory depression: acute and delayed pulmonary responses following aerosolized fentanyl exposure

**DOI:** 10.1093/toxsci/kfag081

**Published:** 2026-07-06

**Authors:** Yesenia Lopez Hernandez, Tanima Chatterjee, Christine M Gross, Lilly Underwood, Kayla F Goliwas, Juan Xavier Masjoan Juncos, Adriana Yndart Arias, Satyanarayana Achanta, Jessy Deshane, Saurabh Aggarwal

**Affiliations:** Department of Cellular and Molecular Medicine, Herbert Wertheim College of Medicine, Florida International University, Miami, FL, 33199, United States; Division of Nephrology, University of Alabama at Birmingham, Birmingham, AL, 35294, United States; Beaufort Memorial Hospital, Beaufort, SC, 29902, United States; Division of Cardiovascular Disease, University of Alabama at Birmingham, Birmingham, AL, 35294, United States; Pulmonary, Allergy, and Critical Care Medicine, University of Alabama at Birmingham, Birmingham, AL, 35294, United States; Anesthesiology and Perioperative Medicine, University of Alabama at Birmingham, Birmingham, AL, 35294, United States; Department of Cellular and Molecular Medicine, Herbert Wertheim College of Medicine, Florida International University, Miami, FL, 33199, United States; Department of Anesthesiology, Duke University School of Medicine, Durham, NC, 27710, United States; Pulmonary, Allergy, and Critical Care Medicine, University of Alabama at Birmingham, Birmingham, AL, 35294, United States; Department of Cellular and Molecular Medicine, Herbert Wertheim College of Medicine, Florida International University, Miami, FL, 33199, United States

**Keywords:** fentanyl, inhalation toxicity, acute lung injury, pulmonary fibrosis, emphysema, IL-1β

## Abstract

Fentanyl is a highly potent synthetic opioid and a major contributor to opioid-related mortality due to central respiratory depression. Inhalational exposure occurs during recreational misuse, polysubstance abuse, occupational handling, and potential chemical threat scenarios. However, whether inhaled fentanyl is associated with pulmonary toxicity independent of central effects remains unclear. Adult male C57BL/6 mice were exposed to aerosolized fentanyl (0.1, 1, or 5 mg/kg; nebulized dose, 5 min) or saline and evaluated 1- and 14 d postexposure. Within 24 h, fentanyl exposure was associated with acute lung injury characterized by alveolar hemorrhage, increased bronchoalveolar lavage protein and inflammatory cells, high lactate dehydrogenase levels, elevated lung wet-to-dry ratio, and reduced arterial oxygenation. Systemic hyperglycemia and increased plasma IL-1β and IL-12p70 were observed, with significant upregulation of caspase-1 activity and IL-1β in lung tissue. Similar IL-1β induction occurred in ex vivo human lung tissue and in vitro human macrophages. At 14 d, delayed dose-dependent remodeling was evident. Low-dose exposure (0.1 mg/kg) produced emphysematous changes, airspace enlargement, and increased mean linear intercept, consistent with emphysema-like changes. Higher doses (1 and 5 mg/kg) were associated with fibrotic remodeling, increased collagen deposition, and fibrotic remodeling. High-dose exposure (5 mg/kg) reduced 14-d survival to 65%. In conclusion, these findings demonstrate that aerosolized fentanyl exposure is associated with acute lung injury and subsequent delayed structural remodeling, accompanied by IL-1β-associated inflammatory signaling. These findings highlight potential pulmonary risks of inhaled fentanyl relevant to substance abuse, occupational safety, emergency response preparedness, and regulatory toxicology risk assessment.

## Graphical Abstract

**Figure:**
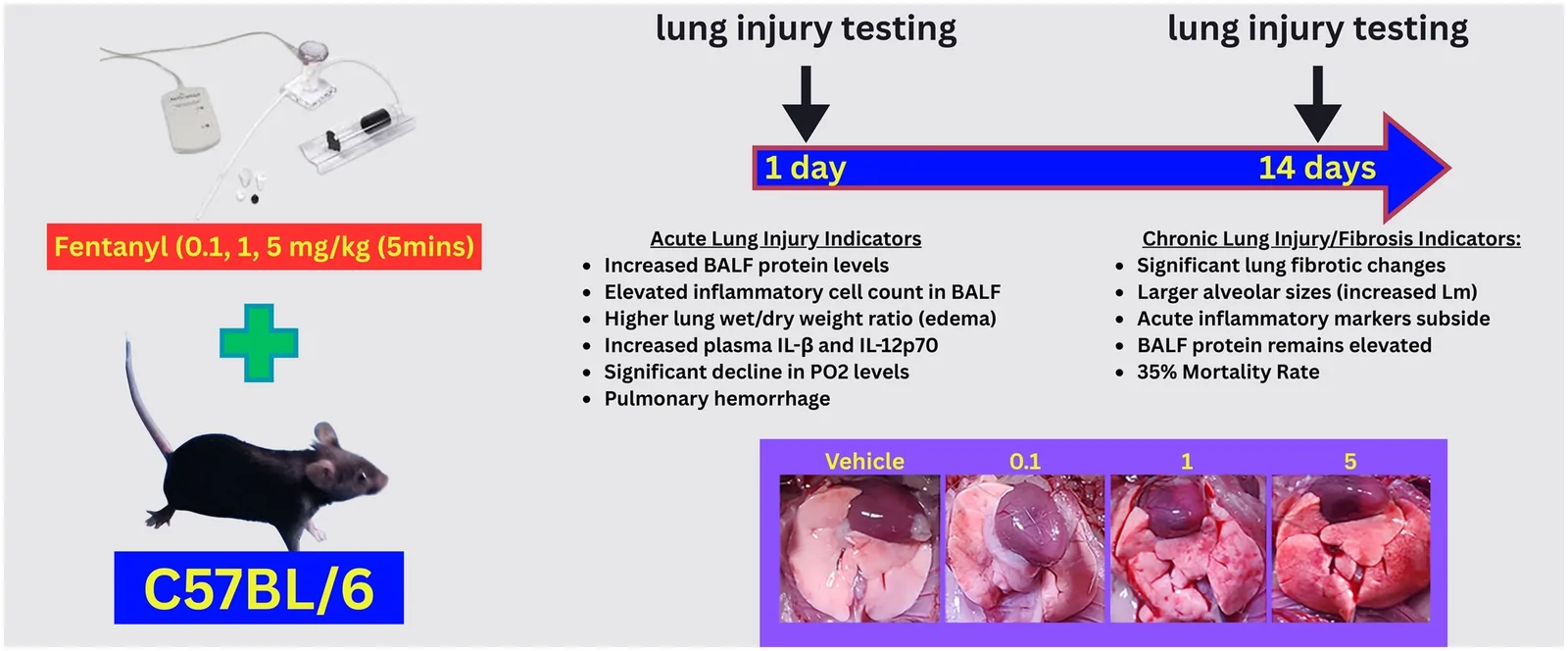

Fentanyl is a highly potent synthetic opioid that plays a central role in the ongoing opioid crisis and is strongly associated with overdose mortality (Blanco et al. 2020; Ciccarone 2021). Its pharmacological effects are mediated primarily through activation of the μ-opioid receptor (MOR), a Gi protein-coupled receptor that inhibits adenylyl cyclase signaling. MORs are widely expressed in the central nervous system and in peripheral tissues of neural and nonneural origin (Dhaliwal and Gupta 2025). In neurons, MOR activation induces membrane hyperpolarization and suppresses neurotransmitter release (Williams et al. 2001; Waldhoer et al. 2004). Clinically, the most critical acute effect of fentanyl is depression of brainstem respiratory centers, resulting in hypoventilation. Additional effects include vagally mediated bradycardia and reduced sympathetic tone, contributing to bronchoconstriction and vasodilation (Rucquoi and Camu 1983; Pattinson 2008).

Due to its high lipid solubility, fentanyl rapidly crosses the blood–brain barrier, producing a rapid onset of action (Henthorn et al. 1999; Bird et al. 2023; Das et al. 2025). Although therapeutic administration occurs via intravenous, transmucosal, or transdermal routes (Grape et al. 2010; Ramos-Matos et al. 2025), inhalation of fentanyl powder has become increasingly prevalent in illicit settings, either intentionally or accidentally. Reports of fentanyl inhalation associated with recreational misuse, polysubstance abuse, and counterfeit pill consumption continue to increase (McAuliffe et al. 2006; Attaway et al. 2021; Bird et al. 2023; Eden et al. 2024; Zhuang et al. 2024; Das et al. 2025). Inhalational exposure may also occur in occupational or emergency-response settings during handling of illicit fentanyl powders or aerosolized materials, although the true pulmonary risks associated with these exposures remain incompletely defined. Prior experimental studies examining inhaled fentanyl have focused predominantly on systemic toxicity and centrally mediated respiratory depression, with limited characterization of pulmonary pathology (Manral et al. 2009). In clinical settings, however, inhaled fentanyl exposure has increasingly been associated with pulmonary complications, including diffuse alveolar hemorrhage and noncardiogenic pulmonary edema (Ruzycki et al. 2016; Tambe et al. 2019; Nangrani et al. 2023; Awad et al. 2024). These observations raise the possibility that inhaled fentanyl exposure may contribute to lung injury beyond its established central nervous system effects.

The lung is highly susceptible to sterile inflammatory injury mediated by innate immune activation, including pathways involving IL-1β and the NLRP3 inflammasome (Liu et al. 2021). Dysregulated IL-1β-associated signaling has been implicated in acute lung injury, pulmonary fibrosis, and chronic obstructive pulmonary disease (Wu et al. 2022). Although inflammatory pathways have not been extensively studied in the context of inhaled fentanyl exposure, clinical reports describing pulmonary edema and diffuse alveolar hemorrhage following fentanyl inhalation suggest that inflammatory mechanisms may contribute to pulmonary injury independently of hypoxia secondary to respiratory depression. Importantly, the pulmonary dose delivered during inhalational fentanyl exposure remains difficult to estimate in both experimental and human settings because aerosol deposition depends on particle size, breathing pattern, airway geometry, and exposure conditions. Therefore, direct extrapolation between nebulized experimental doses and human exposure scenarios should be interpreted cautiously.

We therefore hypothesized that aerosolized fentanyl exposure would be associated with pulmonary inflammatory injury and structural remodeling. Using a controlled murine inhalation model, we evaluated acute and delayed pulmonary responses following aerosolized fentanyl exposure, including inflammatory signaling, barrier dysfunction, gas exchange abnormalities, histopathology, and survival. In addition, we examined caspase-1 activity in murine lungs and IL-1β expression in human macrophages and ex vivo human lung tissue to explore the translational relevance of fentanyl-associated inflammatory responses. These studies identify the lung as a potential target of inhaled fentanyl exposure and provide a framework for future mechanistic investigations relevant to substance misuse, occupational safety, emergency preparedness, and toxicological risk assessment of aerosolized opioids.

## Materials and methods

### Animals and ethical approval

All procedures were approved by the Institutional Animal Care and Use Committee of the University of Alabama at Birmingham and the Florida International University and performed in AAALAC-accredited facilities in accordance with NIH guidelines. Male C57BL/6J mice (7 to 8 wk; The Jackson Laboratory) were housed at 23 ± 1°C under a 12-h light/dark cycle with standard chow (Teklad 2918, Envigo) and water ad libitum. Animals were randomly assigned to treatment groups using a computer-generated sequence. Investigators performing outcome assessments and histological quantification were blinded to treatment allocation. Sample sizes were determined based on prior pilot data and published variability in lung injury endpoints to achieve ≥80% power at α = 0.05.

### Aerosolized fentanyl exposure

Fentanyl citrate was dissolved in sterile saline to deliver 0.1, 1, or 5 mg/kg body weight in a total volume of 1 ml. Aerosols were generated using an Aeroneb Lab Micropump Nebulizer (Kent Scientific), producing particles of 2.5 to 4.0 µm. Conscious mice were placed in a nose-cone exposure chamber, permitting directed inhalation. Aerosol flow was maintained at 0.2 ml/min; 1 ml was delivered over ∼5 min. The reported fentanyl doses (0.1, 1, or 5 mg/kg) represent the nominal nebulized dose relative to body weight and do not represent directly measured inhaled or deposited lung dose. Actual pulmonary deposition may vary depending on respiratory pattern, aerosol distribution, and airway deposition efficiency. To compensate for ∼0.2 ml system dead volume, 20% additional solution was included. Control animals received aerosolized saline under identical conditions. For end-of-study procedures, at 1 h, 1 d, or 14 d postexposure, mice were anesthetized with ketamine (100 mg/kg) and xylazine (10 mg/kg, i.p.), and then euthanized by exsanguination via the abdominal aorta and organ removal. Lungs were perfused with ice-cold PBS/EDTA, excised, snap-frozen, and stored at 80°C.

### Plasma isolation and analysis

Blood was centrifuged (2,000 × g, 10 min, 4°C) to obtain plasma. Fentanyl concentrations were quantified using a commercial ELISA kit (Neogen, Catalog No. 131519). A panel of 10 cytokines was quantified in 75 μl plasma using the V-PLEX Proinflammatory Panel 1 mouse kit (K15048D).

### Bronchoalveolar lavage fluid

Bronchoalveolar lavage fluid (BALF) was collected by instillation and recovery of 1 ml sterile saline via tracheal cannulation (0.7 to 0.9 ml recovered). Samples were centrifuged (3,000 × g, 10 min at 4°C). Cell pellets were processed immediately following centrifugation for total cell counting using a hemocytometer. Cell morphology was routinely inspected microscopically following processing. Supernatants were used for protein quantification. Hemoglobin concentration in BALF was determined spectrophotometrically at 540 nm as an index of alveolar–capillary barrier disruption.

### Lactate dehydrogenase assay

BALF supernatants were analyzed for lactate dehydrogenase (LDH) activity as a marker of cellular injury using a commercial colorimetric assay kit (Cat No. 11644793001, Roche) according to the manufacturer’s instructions. Briefly, BALF samples were incubated with LDH reaction mixture in 96-well plates, and absorbance was measured at 490 nm. LDH activity was expressed relative to vehicle (saline)-treated controls.

### Caspase-1 inflammasome assay

Caspase-1 activity was measured in mouse lung tissue homogenate using the Caspase-Glo 1 Inflammasome Assay (Cat No. G9951, Promega) according to the manufacturer’s instructions.

### Lung wet-to-dry ratio

Pulmonary edema was assessed 1 or 14 d after exposure. Lungs were weighed immediately (wet weight), dried at 80°C for 36 h, and reweighed. The wet-to-dry ratio was calculated as wet weight/dry weight.

### Arterial blood gas analysis

Mice were anesthetized with isoflurane (5% induction, 2% maintenance). Arterial blood was collected from the abdominal aorta into heparinized syringes and analyzed immediately using an Element POC analyzer (Heska).

### Histology and morphometry

At day 14, lungs were inflated with 10% neutral-buffered formalin at 25 cm H_2_O for 1 h, fixed for 48 h, paraffin-embedded, sectioned, and stained with hematoxylin and eosin or Masson’s trichrome. Airspace enlargement was quantified by mean linear intercept (Lm) analysis using standardized morphometric methods adapted from established protocols (Hsia et al. 2010). Twenty random nonoverlapping parenchymal fields per section were analyzed at ×20 magnification by a blinded observer. Fields containing large conducting airways, major blood vessels, or pleural borders were excluded. A grid overlay was applied using standardized imaging software parameters, and intercepts between alveolar walls and test lines were counted according to established morphometric criteria. Mean linear intercept values were calculated as the total length of test lines divided by the number of alveolar wall intercepts. Total collagen content was determined by hydroxyproline assay (Sigma-Aldrich, MAK008).

### Cell culture

THP-1 cells (catalog no. TIB-202) were purchased from ATCC and cultured in RPMI-1640 medium that was supplemented with 10% FBS, 2 mM L-glutamine, 100 IU/ml penicillin, and 100 µg/ml streptomycin. The cells were incubated at 37°C, with 95% humidity and 5% CO_2_. For macrophage polarization, THP-1 monocytes were treated with PMA in RPMI media (10 ng/ml, 24 h). The media was then changed, and cells were allowed to incubate for an additional 48 h in RPMI medium. THP-1-derived macrophages were exposed to fentanyl (1 to 750 µM) for 24 h. The concentration range was selected to encompass potential local exposure conditions following pulmonary aerosol deposition and was not intended to directly model circulating plasma fentanyl concentrations observed clinically.

### Ex vivo human lung culture

Uninvolved deidentified human lung tissue was obtained from consented patients undergoing resection through the UAB Tissue Procurement Shared Facility, in accordance with institutional IRB approval. Three-millimeter tissue cores were generated and placed in perfusion bioreactors (6 cores/chamber; 4 to 8 cultures per specimen) (Ahmad et al. 2024; Goliwas et al. 2025). After 1 to 3 d of stabilization, tissues were exposed to fentanyl at concentrations selected to approximate relative low-, intermediate-, and high-exposure conditions corresponding to the in vivo aerosol exposure paradigm. or vehicle for 24 h. Tissue was subsequently processed for RNA isolation and cytokine analysis.

### Gene expression

Total RNA was extracted using TRIzol and purified (RNeasy, Qiagen). RNA purity and concentration were verified spectrophotometrically. One microgram of RNA was reverse-transcribed, and quantitative PCR was performed using SYBR Green chemistry (Applied Biosystems) on a Bio-Rad CFX Opus 96 system. IL-1β expression was assessed in mouse lung tissue, fentanyl-treated THP-1 cells, and human lung cultures. β-actin served as the reference gene. Relative expression was calculated using the 2^−ΔΔCt method. Technical duplicates were run for each sample.

### Survival studies

Following exposure, mice were monitored continuously. Mortality was recorded. Body weight was measured serially to evaluate animal distress. Survival curves were generated using the Kaplan–Meier method and compared using the log-rank test. Humane endpoints such as loss of 20% body weight and severe lethargy were predefined, and animals meeting criteria were euthanized and recorded as mortality events.

### Statistical analysis

Data are presented as mean ± SEM. Statistical analyses were performed using GraphPad Prism. Normality was assessed using the Shapiro–Wilk test. Comparisons among ≥2 groups were performed using 1-way ANOVA with Tukey’s post hoc testing where appropriate. Survival differences were analyzed by the log-rank test. Two-tailed *P* < 0.05 was considered statistically significant. For 14-d analyses, only surviving animals were included in endpoint measurements.

## Results

### Pulmonary delivery of aerosolized fentanyl results in sustained systemic exposure

**Fig. 1.**
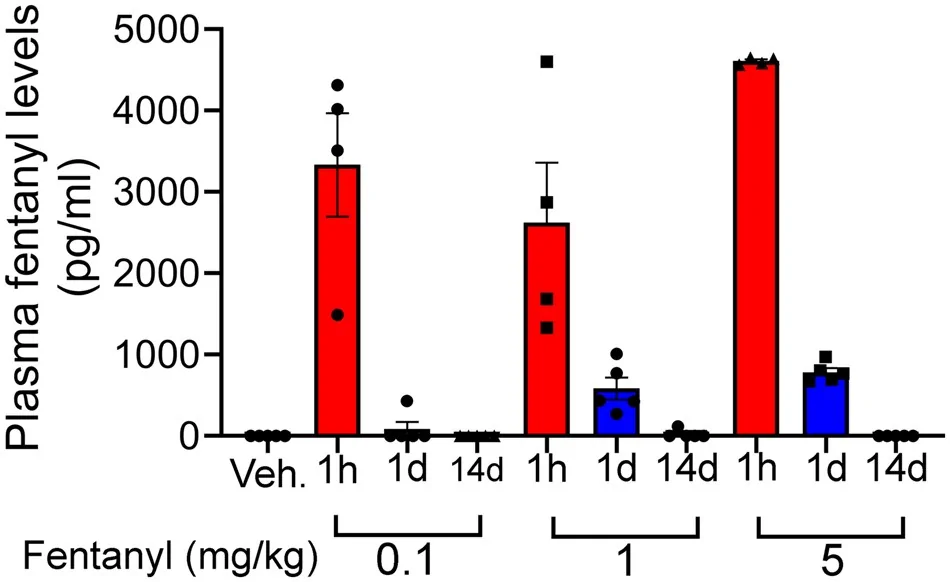
Plasma fentanyl levels. Adult male C57BL/6 mice were nebulized with saline (vehicle) or fentanyl (0.1, 1, or 5 mg/kg BW) for 5 min. The data show plasma levels of fentanyl at 1 h, 1 d, and 14 d postexposure (n = 4 to 5). Individual values and means±SEM.

To confirm effective pulmonary delivery and systemic absorption, plasma fentanyl concentrations were measured at 1 h, 24 h, and 14 d following a 5-min aerosol exposure (0.1, 1, or 5 mg/kg). All fentanyl doses produced significant elevations in plasma levels at 1 h compared with saline controls (Fig. 1). At 1 d postexposure, fentanyl remained detectable in the 1 and 5 mg/kg groups, whereas levels in the 0.1 mg/kg group were below detection limits (Fig. 1). These findings confirm that inhaled fentanyl achieves systemic exposure following pulmonary deposition.

### Inhaled fentanyl is associated with acute pulmonary barrier disruption and hemorrhagic injury

**Fig. 2.**
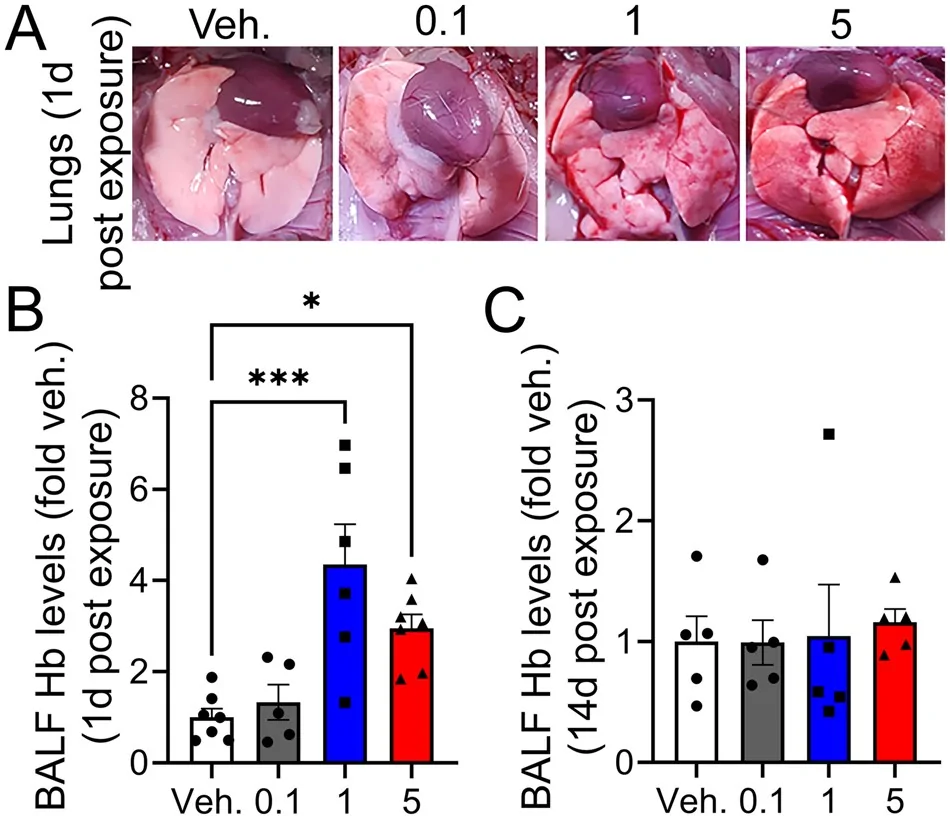
Pulmonary hemorrhage postfentanyl exposure. Adult male C57BL/6 mice were nebulized with saline (vehicle) or fentanyl (0.1, 1, or 5 mg/kg, 5 min). (A) Macroscopic images of naïve lungs preperfusion, 1-d postfentanyl exposure (n = 4 to 5). (B) BALF hemoglobin levels 1-d and (C) 14-d postfentanyl exposure. (n = 5 to 7) Individual values and means±SEM. *P < 0.05 versus vehicle (1-way ANOVA followed by Tukey’s post hoc testing).

Consistent with the hypothesis that fentanyl exposure is associated with injury to the lung parenchyma, marked features of acute lung injury were observed 1 d after exposure. Gross inspection of naïve lungs preperfusion demonstrated visible pulmonary hemorrhage in mice exposed to 1 and 5 mg/kg fentanyl (Fig. 2A). BALF hemoglobin concentrations were significantly elevated in these groups at day 1 (Fig. 2B), indicating alveolar–capillary barrier disruption. Hemorrhagic indices returned to baseline by day 14 in the animals that survived (Fig. 2C).

**Fig. 3.**
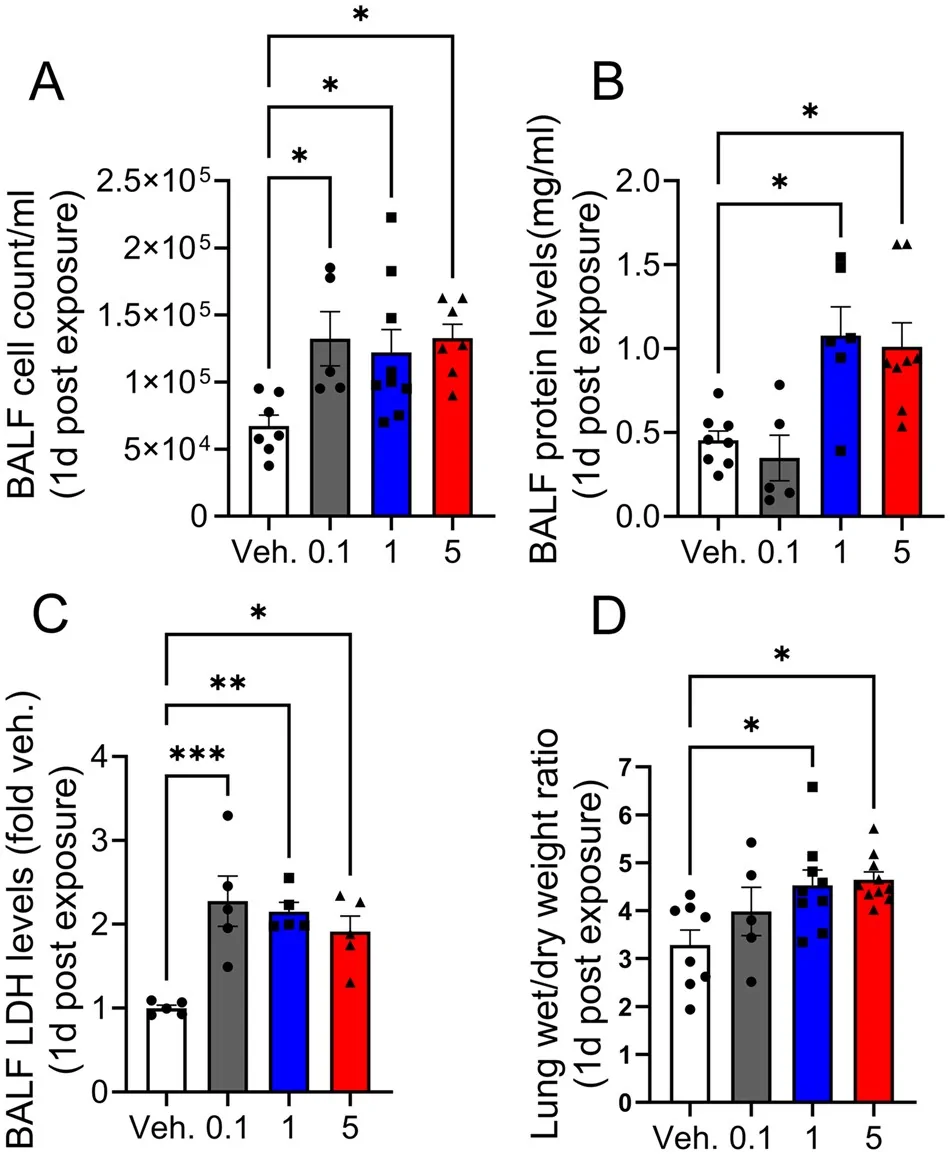
Acute lung injury: Adult male C57BL/6 mice were nebulized with saline (vehicle) or fentanyl (0.1, 1, or 5 mg/kg, 5 min). Fentanyl exposure increased BALF inflammatory cells (A), proteins (B), LDH activity (C), and lung wet/dry weight ratio (D) 1-d postexposure. (n = 5 to 9) Individual values and means±SEM. *P < 0.05 versus vehicle (1-way ANOVA followed by Tukey’s post hoc testing).

Fentanyl inhalation also increased inflammatory and permeability markers at 24 h. BALF inflammatory cell counts were significantly elevated at all tested doses (0.1, 1, and 5 mg/kg) (Fig. 3A). BALF protein concentrations (Fig. 3B) were increased relative to saline controls. BALF LDH activity was significantly increased following fentanyl exposure, consistent with increased pulmonary cellular injury and membrane disruption (Fig. 3C). In addition, lung wet-to-dry weight ratios were elevated in the 1 and 5 mg/kg groups (Fig. 3D), consistent with pulmonary edema. Functionally, arterial blood gas analysis revealed a significant reduction in arterial PO_2_ at 24 h following fentanyl exposure (Table 1), accompanied by systemic hyperglycemia. Together, these data demonstrate acute fentanyl-induced pulmonary barrier dysfunction with impaired gas exchange.

**Table 1.** Arterial blood gases (ABG).

ABG (mean ± SD)	Vehicle	Fentanyl (0.1 mg/kg)	Fentanyl (1 mg/kg)	Fentanyl (5 mg/kg)
**pO_2_**	88.6 ± 6.4	80.4 ± 10	72.9 ± 4.7*	72.7 ± 10.6*
**cSO_2_**	92.3 ± 3.3	93.0 ± 2.5	90.5 ± 2.5	90.0 ± 3.7
**pCO_2_**	56.3 ± 7.7	61.9 ± 6.0	60.7 ± 9.6	60.2 ± 2.7
**HCO_3_**	26.7 ± 2.4	28.0 ± 2.0	25.5 ± 3.4	25.2 ± 1.9
**pH**	7.24 ± 0.1	7.26 ± 0.0	7.23 ± 0.0	7.23 ± 0.0
**Na**	146.2 ± 3.1	146.8 ± 2.6	148.8 ± 5.8	147.6 ± 2.7
**K**	4.0 ± 0.6	3.9 ± 0.2	4.2 ± 0.5	4.0 ± 0.4
**Cl−**	110.2 ± 4.0	109.4 ± 2.5	113.8 ± 5.6	112.4 ± 4.7
**Ca^2+^**	1.2 ± 0.0	1.3 ± 0.0	1.2 ± 0.2	1.3 ± 0.1
**AGAP**	15.4 ± 2.9	14.0 ± 3.0	14.0 ± 2.3	14.2 ± 3.7
**Lactate**	1.4 ± 0.7	0.9 ± 0.2	1.0 ± 0.3	1.5 ± 0.9
**BUN**	24.8 ± 3.2	21.6 ± 3.2	23.0 ± 2.4	20.6 ± 3.3
**Glucose**	305.8 ± 56.5	446.6 ± 36*	433.0 ± 75*	421.4 ± 55*
**HCT**	34.4 ± 3.5	35.4 ± 0.5	35.2 ± 2.9	33.4 ± 0.5

Adult male C57BL/6 mice were nebulized with saline (vehicle) or fentanyl (0.1, 1, or 5 mg/kg, 5 min). Fentanyl decreased arterial PO_2_ and blood glucose in mice 1-d postexposure. (n = 5) means±SEM. *P < 0.05 versus vehicle (1-way ANOVA followed by Tukey’s post hoc testing).

### Fentanyl enhances IL-1β-associated innate immune signaling in vivo and in human lung systems

**Fig. 4.**
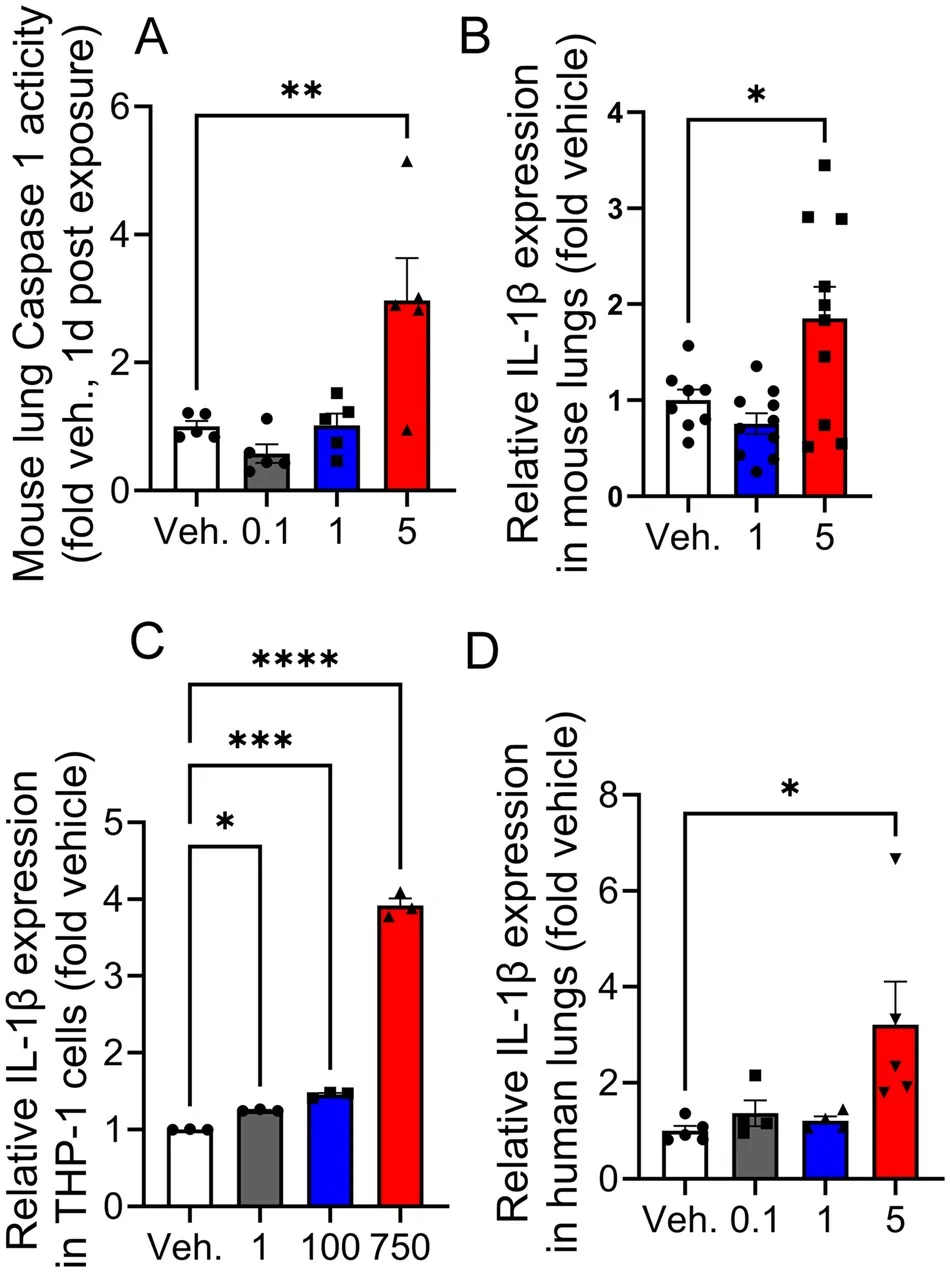
Caspase-1 activity and IL-1β expression. Adult male C57BL/6 mice were nebulized with saline (vehicle) or fentanyl (0.1, 1, or 5 mg/kg, 5 min). (A, B) Exposure to 5 mg/kg of fentanyl increased lung caspase-1 levels and IL-1β mRNA at 1 d (n = 5 to 10). (C) Differentiated THP-1 cells exposed to fentanyl (0.1, 1, or 750 µM) showed increased IL-1β (n = 3). (D) Human lung tissue exposed to fentanyl (5 mg/kg) also showed increased IL-1β at 1 d (n = 4 to 5). Data are mean±SEM; *P < 0.05 versus vehicle (1-way ANOVA with Tukey’s post hoc test).

Given the proposed role of inflammasome-associated pathways in sterile lung injury, inflammatory cytokines were assessed 1 d after exposure. Plasma IL-1β and IL-12p70 were significantly elevated following fentanyl inhalation (Table 2), indicating systemic activation of proinflammatory signaling. In lung tissue, caspase-1 activity (Fig. 4A) and IL-1β mRNA expression (Fig. 4B) were significantly increased in mice exposed to 5 mg/kg fentanyl compared with the vehicle-treated animals, consistent with activation of IL-1β-associated innate immune responses within the pulmonary parenchyma. To determine whether fentanyl directly modulates macrophage inflammatory signaling, differentiated THP-1 macrophages were treated with fentanyl (1 to 750 µM) for 24 h. Although the in vitro fentanyl concentrations exceed typical circulating plasma levels observed clinically, they were selected to approximate potential local tissue exposure following pulmonary deposition. IL-1β mRNA expression was significantly increased compared with vehicle-treated cells (Fig. 4C). Similarly, ex vivo human lung tissue exposed to fentanyl for 24 h demonstrated dose-dependent induction of IL-1β expression, reaching significance at 5 mg/kg equivalent concentration (Fig. 4D). Collectively, these findings demonstrate that fentanyl exposure enhances IL-1β expression in murine lung, human macrophages, and intact human lung tissue, supporting activation of innate immune signaling pathways consistent with inflammasome-associated responses.

**Table 2.** Plasma cytokine profile.

Plasma cytokines (pg/ml)	Vehicle	Fentanyl (0.1 mg/kg)	Fentanyl (1 mg/kg)	Fentanyl (5 mg/kg)
**TNF-α**	5.2 ± 1.3	5.5 ± 1.3	5.7 ± 1.5	5.7 ± 0.7
**KC/GRO**	62.1 ± 59.7	66.5 ± 22.1	70. ± 32.6	68.5 ± 24.1
**IL-6**	26.7 ± 16.2	17.2 ± 6.7	22.5 ± 7.0	27.3 ± 11.3
**IL-5**	11.9 ± 4.7	11.6 ± 5.6	12.6 ± 2.6	13.3 ± 3.1
**IL-2**	1.2 ± 0.6	1.2 ± 0.5	1.5 ± 0.7	0.4 ± 0.1
**IL-1β**	0.6 ± 0.1	0.6 ± 0.2	0.8 ± 0.2	1.3 ± 0.4*
**IL-12p70**	26.8 ± 7.1	31.2 ± 8.7	40.8 ± 7.8*	43.7 ± 5.5*
**IL-10**	5.2 ± 1.9	5.6 ± 1.4	6.1 ± 2.1	6.2 ± 2.9
**IFN-γ**	0.2 ± 0.1	0.4 ± 0.2	0.5 ± 0.4	0.3 ± 0.2

Adult male C57BL/6 mice were nebulized with saline (vehicle) or fentanyl (0.1, 1, or 5 mg/kg, 5 min). Fentanyl increased plasma IL-1β and IL-12p70 in mice 1-d postexposure. (n = 5) means±SEM. *P < 0.05 versus vehicle (1-way ANOVA followed by Tukey’s post hoc testing).

### Divergent chronic remodeling following fentanyl exposure

**Fig. 5.**
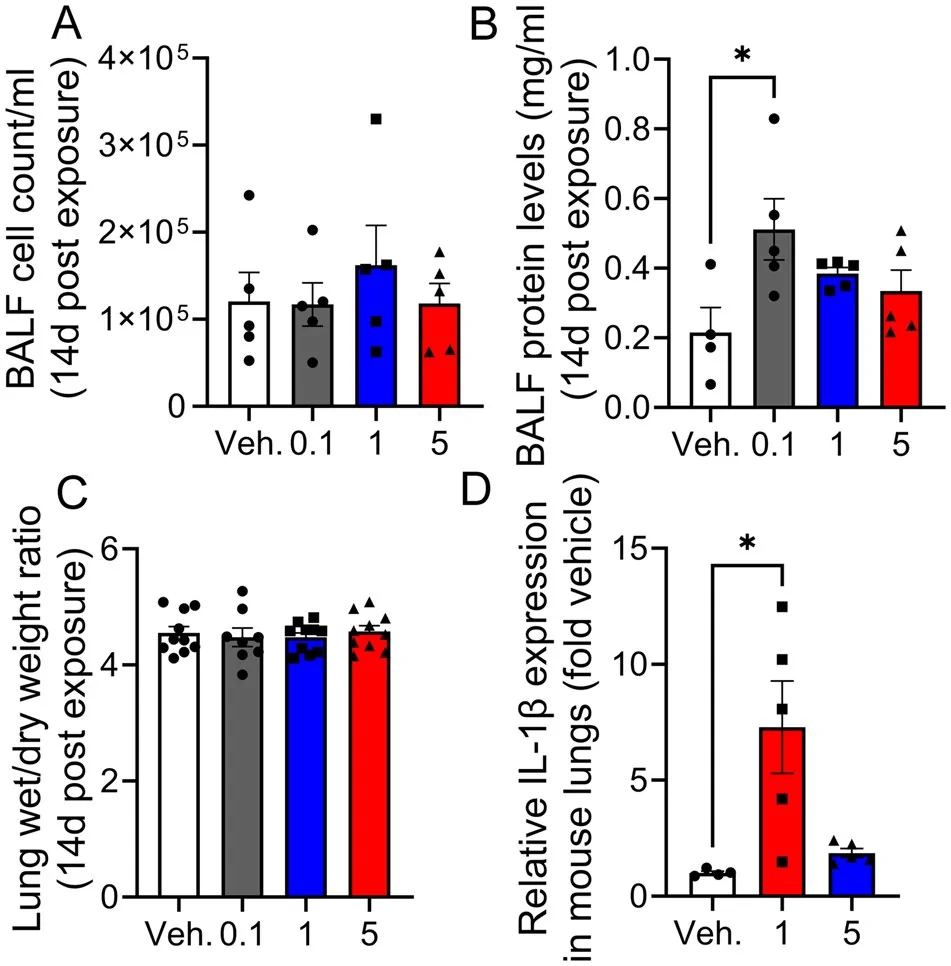
Chronic lung injury. Adult male C57BL/6 mice were nebulized with saline (vehicle) or fentanyl (0.1, 1, or 5 mg/kg, 5 min). The analysis of (A) BALF cell count (n = 5), (B) BALF protein levels (n = 4 to 5), and (C) lung wet/dry weight ratio (n = 8 to 10), showed a resolution of acute lung injury parameters 14-d postfentanyl exposure. (D) However, the lung IL-1β mRNA expression was elevated 14-d postfentanyl (1 mg/kg) exposure. Individual values and means±SEM. *P < 0.05 versus vehicle (1-way ANOVA followed by Tukey’s post hoc testing).

At 14 d postexposure, several acute inflammatory parameters had resolved. BALF inflammatory cell counts and lung wet-to-dry ratios returned to control levels (Fig. 5A and C). However, BALF protein remained elevated in mice exposed to 0.1 mg/kg fentanyl (Fig. 5B), and persistent elevation of lung IL-1β expression was observed in the 1 mg/kg group (Fig. 5D), suggesting ongoing low-grade inflammatory signaling.

**Fig. 6.**
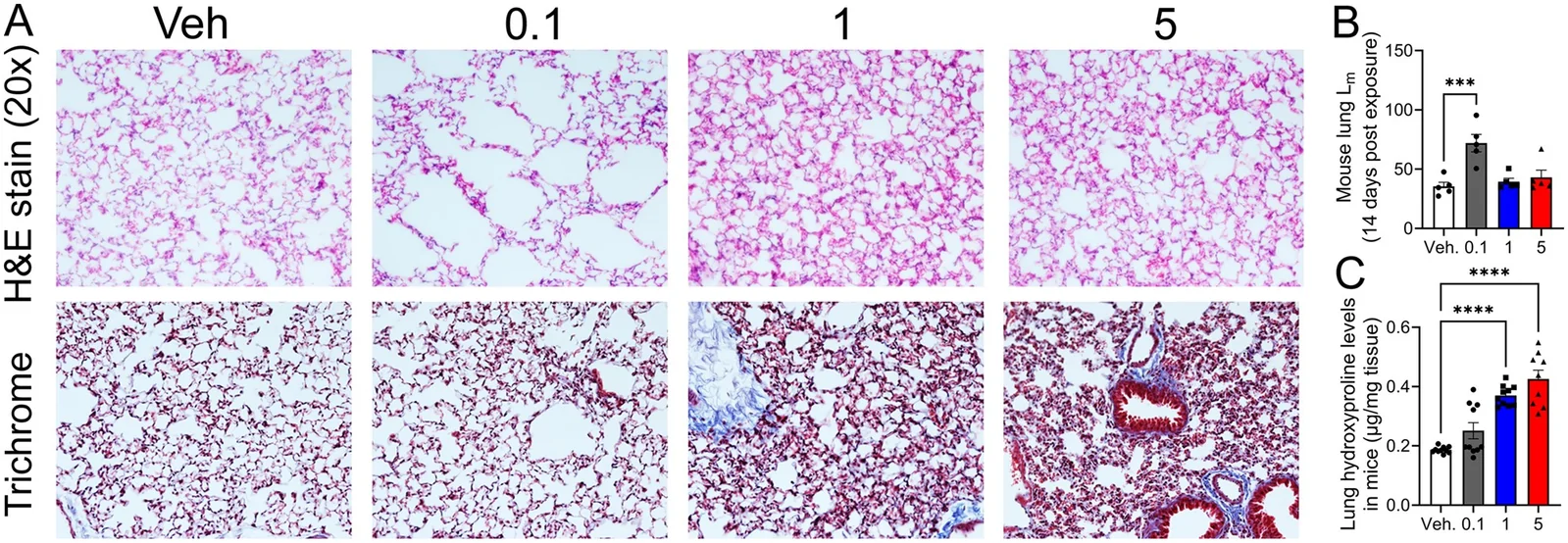
Pulmonary remodeling. Adult male C57BL/6 mice were nebulized with saline (vehicle) or fentanyl (0.1, 1, or 5 mg/kg, 5 min). (A) Top panel: H&E staining of lungs and (B) mean linear intercept (Lm) shows airspace enlargement consistent with emphysema-like changes 14-d postexposure to 0.1 mg/kg of fentanyl. (n = 5) (A) Bottom panel: Trichrome staining and (C) lung hydroxyproline levels show fibrotic remodeling and increased collagen deposition in mice exposed to 1 or 5 mg/kg of fentanyl. (n = 5 to 10). Individual values and means±SEM. *P < 0.05 versus vehicle (1-way ANOVA followed by Tukey’s post hoc testing).

Histological analysis 14 d postexposure revealed dose-dependent divergence in chronic remodeling patterns. Mice exposed to the low dose (0.1 mg/kg) exhibited alveolar wall thinning and airspace enlargement on H&E staining (Fig. 6A). Quantitative morphometry confirmed a significant increase in mean linear intercept (Lm) (Fig. 6B), consistent with emphysematous remodeling. In contrast, higher doses (1 and 5 mg/kg) were associated with fibrotic remodeling. Lung hydroxyproline content was significantly elevated in these groups (Fig. 6C), indicating increased collagen deposition. Enhanced trichrome staining corroborated extracellular matrix accumulation (Fig. 6A). These findings indicate that inhaled fentanyl promotes dose-dependent chronic structural remodeling, characterized by emphysematous changes at low doses and fibrotic remodeling at higher doses.

### High-dose fentanyl reduces 14-d survival

**Fig. 7.**
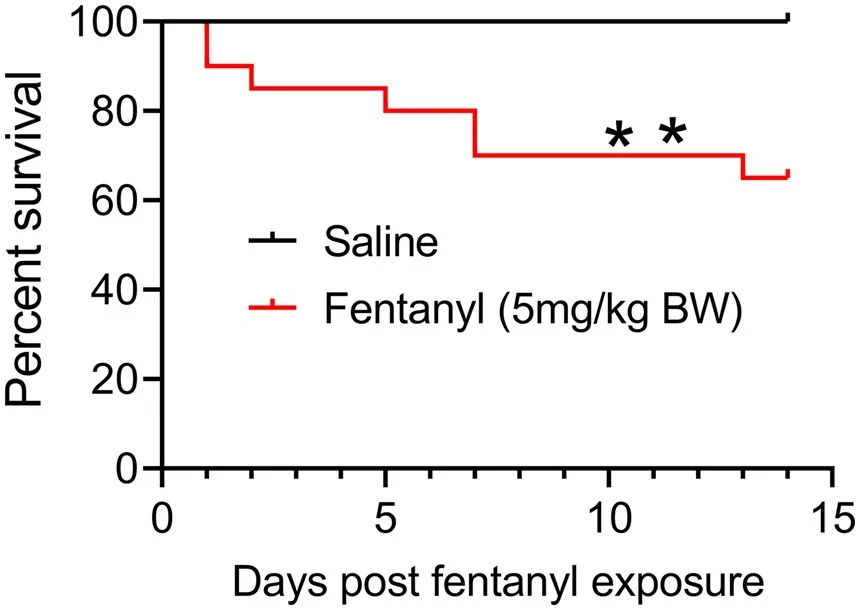
Mouse survival. Adult male C57BL/6 mice were nebulized with saline (vehicle) or fentanyl (5 mg/kg, 5 min). The Kaplan–Meier curve shows that exposure to fentanyl reduced 14-d survival to 65% (n = 20).

Kaplan–Meier analysis demonstrated that a single 5-min exposure to 5 mg/kg aerosolized fentanyl reduced 14-d survival to 65% (Fig. 7). No mortality was observed in saline-treated animals. These results indicate that high-dose inhalational exposure produces sustained systemic and pulmonary toxicity associated with increased mortality.

## Discussion

This study suggests that acute exposure to aerosolized fentanyl is associated with pulmonary parenchymal injury in rodents. Mice were exposed to 0.1, 1, and 5 mg/kg nebulized doses of fentanyl. Within 24 h, mice exhibited hallmark features of acute lung injury, including alveolar hemorrhage, elevated BALF protein, inflammatory cell infiltration, increased BALF LDH activity, and pulmonary edema. Lung wet-to-dry ratios and arterial hypoxemia further confirmed compromised alveolar–capillary barrier function. Systemic absorption of fentanyl was confirmed, with detectable plasma levels up to 24 h postexposure, consistent with prior pharmacokinetic profiles reported in other preclinical inhalation models and human studies (Mather et al. 1998; Manral et al. 2009; Alishlash et al. 2021; Shelton and Nicholson 2022; Bansari et al. 2024). Beyond acute toxicity, we observed dose-dependent delayed structural remodeling. By 14 d postexposure, low-dose fentanyl (0.1 mg/kg) was associated with airspace enlargement and increased mean linear intercept, consistent with emphysema-like changes, whereas higher doses (1 and 5 mg/kg) were associated with fibrotic changes, reflected by increased collagen deposition and fibrotic remodeling. High-dose exposure was also associated with reduced survival (65% at 14 d). Collectively, these findings suggest a progression from acute injury to delayed structural remodeling following inhaled fentanyl exposure.

Our results support the concept that fentanyl exerts direct pulmonary toxicity and suggest that aerosolized fentanyl exposure may contribute to pulmonary toxicity, although the relative contributions of direct pulmonary effects versus systemic opioid effects cannot be fully distinguished in the current study (Gao et al. 2025). Although opioids are known to cause hypoventilation via μ-opioid receptor activation, additional direct effects on pulmonary tissue have been described (Safrai 2007; Ruzycki et al. 2016; Tambe et al. 2019; Nangrani et al. 2023). Intravenous fentanyl can induce chest wall rigidity, impairing ventilation and contributing to hypoxemia and hypercarbia. Importantly, we did not directly measure respiratory rate, ventilation, or sedation, and therefore cannot exclude contributions from altered breathing or systemic hypoxemia to the observed lung injury. Future studies incorporating respiratory monitoring or pharmacologic blockade (e.g. naloxone) will be important to clarify these mechanisms. The observed inflammatory response was associated with increased caspase-1 activity in the mouse lung and elevated IL-1β in plasma and lung tissue. Similar IL-1β induction was observed in THP-1-derived macrophages and ex vivo human lung tissue. These findings are consistent with IL-1β-associated inflammatory signaling pathways that have been implicated in sterile lung injury (Liu et al. 2021; Wu et al. 2022). However, comprehensive evidence of inflammasome activation (e.g. NLRP3 assembly or IL-18 maturation) was not assessed, and therefore, inflammasome involvement should be considered putative.

The precise mechanisms of opioid-induced lung injury remain incompletely understood (Alishlash et al. 2021). However, emerging evidence suggests these mechanisms largely operate independently of μ-opioid receptor activation, involving a complex interplay of increased pulmonary epithelial and vascular endothelial permeability, inflammatory cell migration, and widespread cytokine release (Shook et al. 1990; Radke et al. 2014; Alishlash et al. 2021). Elevated cell-free hemoglobin in BALF suggests alveolar–capillary barrier disruption and subsequent heme-mediated inflammatory signaling (Matalon et al. 2015; Aggarwal et al. 2016;   Chatterjee et al. 2023). Fentanyl may also influence vascular tone and endothelial permeability through mechanisms that are not fully dependent on μ-opioid receptor activation. In addition, opioid-associated hypoventilation or airway obstruction could contribute indirectly to lung injury through hypoxia or negative-pressure mechanisms (Johnson II and Anne 2022; Unger and Martin 2023). These possibilities highlight the complexity of disentangling direct pulmonary effects from systemic physiological responses in inhalation exposure models.

Clinically, these findings are relevant to occupational, recreational, and emergency scenarios involving aerosolized fentanyl and underscore an urgent need to understand its direct pulmonary toxicity, distinct from its systemic CNS effects (McAuliffe et al. 2006; Attaway et al. 2021; Eden et al. 2024; Zhuang et al. 2024; Das et al. 2025). Recent clinical case reports have brought to light a concerning pattern of direct alveolar damage caused by inhaled fentanyl, leading to severe noncardiogenic pulmonary edema and diffuse alveolar hemorrhage (Ruzycki et al. 2016; Tambe et al. 2019; Nangrani et al. 2023). A recent retrospective case series further solidified these clinical observations, detailing 8 cases of diffuse alveolar hemorrhage directly attributable to fentanyl inhalation (Awad et al. 2024). Moreover, several autopsy findings in opioid overdose cases, including those involving fentanyl, have corroborated the presence of diffuse alveolar hemorrhage (Chaturvedi et al. 1990; Safrai 2007; Nangrani et al. 2023). Our data extend these observations by providing a controlled preclinical model that captures both acute inflammatory injury and subsequent chronic remodeling, including emphysematous and fibrotic changes.

Although previous studies have investigated the effects of inhaled fentanyl in animal models, primarily focusing on acute mortality due to central respiratory depression, our investigation uniquely distinguishes itself. Notably, one study demonstrated acute inflammatory changes in mouse lungs following a 4-h continuous exposure to aerosolized fentanyl, indicating direct lung effects (Manral et al. 2009). They found that mortality was dose-dependent, likely due to reduced central drive to respiratory muscles. External observation showed no pulmonary or airway irritation, but the mice’s lungs were not examined directly for injury. Building upon this foundational understanding, our study stands as the first to comprehensively characterize the progression of inhaled fentanyl-associated lung injury from acute inflammation and hemorrhage to significant delayed fibrotic changes within the lung parenchyma in an experimental animal model. This detailed longitudinal assessment provides a crucial bridge between acute toxicological effects and long-term pathological outcomes observed clinically.

Several limitations should be considered. First, aerosol dosimetry was defined in terms of nebulized dose, and the actual inhaled or deposited lung dose was not directly measured. Second, respiratory parameters such as ventilation and oxygen saturation were not continuously monitored, limiting the ability to distinguish pulmonary injury from systemic opioid effects. Third, BALF cellular analysis was limited to total cell counts without differential characterization. Although total BALF cell recovery was consistent across groups, the centrifugation conditions used during BALF processing may have affected fragile cell populations. Fourth, IL-1β was used as an inflammatory marker, but causal involvement in lung injury was not tested. Finally, survival at the highest dose introduces potential survivor bias in the 14-d analyses, as animals that survived may not represent the full spectrum of injury.

In summary, aerosolized fentanyl exposure is associated with acute lung injury characterized by alveolar–capillary barrier disruption, hemorrhage, and IL-1β-associated inflammatory responses. These changes are followed by dose-dependent delayed structural remodeling, including airspace enlargement at lower exposure and fibrotic remodeling at higher exposure levels, along with increased mortality at the highest dose. These findings identify the lung as a potential target of inhaled fentanyl exposure and underscore the need for further mechanistic studies to define the pathways involved and their relevance to human health.
